# Systemic Inflammation in Pregnant Women With Latent Tuberculosis Infection

**DOI:** 10.3389/fimmu.2020.587617

**Published:** 2021-01-27

**Authors:** Shilpa Naik, Mallika Alexander, Pavan Kumar, Vandana Kulkarni, Prasad Deshpande, Su Yadana, Cheng-Shiun Leu, Mariana Araújo-Pereira, Bruno B. Andrade, Ramesh Bhosale, Subash Babu, Amita Gupta, Jyoti S. Mathad, Rupak Shivakoti

**Affiliations:** ^1^ Byramjee-Jeejeebhoy Government Medical College-Johns Hopkins University Clinical Research Site, Pune, India; ^2^ Department of Obstetrics and Gynecology, Byramjee Jeejeebhoy Government Medical College, Pune, India; ^3^ International Center for Excellence in Research, National Institutes of Health, National Institute for Research in Tuberculosis, Chennai, India; ^4^ Department of Epidemiology, Columbia University Mailman School of Public Health, New York, NY, United States; ^5^ Department of Biostatistics, Columbia University Mailman School of Public Health, New York, NY, United States; ^6^ Instituto Goncalo Moniz, Fundação Oswaldo Cruz, Salvador, Brazil; ^7^ Multinational Organization Network Sponsoring Translational and Epidemiological Research, Fundação José Silveira, New York, NY, Brazil; ^8^ Faculdade de Medicina, Universidade Federal da Bahia, Salvador, Brazil; ^9^ Curso de Medicina, Faculdade de Tecnologia e Ciências, Salvador, Brazil; ^10^ Escola de Medicina, Universidade Salvador (UNIFACS), Laureate International Universities, Salvador, Brazil; ^11^ Curso de Medicina, Escola Bahiana de Medicina e Saúde Pública (EBMSP), Salvador, Brazil; ^12^ Department of Medicine, Johns Hopkins University School of Medicine, Baltimore, MD, United States; ^13^ Department of Medicine, Weill Cornell Medical College, New York, NY, United States

**Keywords:** latent tuberculosis infection, tuberculosis disease, inflammation, pregnancy, cytokines, LTBI, TB

## Abstract

**Background:**

Recent studies in adults have characterized differences in systemic inflammation between adults with and without latent tuberculosis infection (LTBI+ *vs*. LTBI−). Potential differences in systemic inflammation by LTBI status has not been assess in pregnant women.

**Methods:**

We conducted a cohort study of 155 LTBI+ and 65 LTBI− pregnant women, stratified by HIV status, attending an antenatal clinic in Pune, India. LTBI status was assessed by interferon gamma release assay. Plasma was used to measure systemic inflammation markers using immunoassays: IFN*β*, CRP, AGP, I-FABP, IFN*γ*, IL-1*β*, soluble CD14 (sCD14), sCD163, TNF, IL-6, IL-17a and IL-13. Linear regression models were fit to test the association of LTBI status with each inflammation marker. We also conducted an exploratory analysis using logistic regression to test the association of inflammatory markers with TB progression.

**Results:**

Study population was a median age of 23 (Interquartile range: 21–27), 28% undernourished (mid-upper arm circumference (MUAC) <23 cm), 12% were vegetarian, 10% with gestational diabetes and 32% with HIV. In multivariable models, LTBI+ women had significantly lower levels of third trimester AGP, IL1β, sCD163, IL-6 and IL-17a. Interestingly, in exploratory analysis, LTBI+ TB progressors had significantly higher levels of IL1*β*, IL-6 and IL-13 in multivariable models compared to LTBI+ non-progressors.

**Conclusions:**

Our data shows a distinct systemic immune profile in LTBI+ pregnant women compared to LTBI− women. Data from our exploratory analysis suggest that LTBI+ TB progressors do not have this immune profile, suggesting negative association of this profile with TB progression. If other studies confirm these differences by LTBI status and show a causal relationship with TB progression, this immune profile could identify subsets of LTBI+ pregnant women at high risk for TB progression and who can be targeted for preventative therapy.

## Introduction

Active tuberculosis (TB) disease elicits host responses characterized by an immune profile that is clearly distinct from healthy individuals (1, 2). As the causative agent *Mycobacterium tuberculosis (Mtb)* is actively replicating during TB disease, it causes constant antigen stimulation from the bacterium that shapes the immune response. In contrast, with latent TB infection (LTBI), *Mtb* is not actively replicating in the host and antigen stimulation with *Mtb* antigens is required to generate *Mtb*-specific immune responses (1). While differences in immunity with *Mtb* antigen stimulation has been extensively studied for active disease or LTBI compared to healthy individuals (1–5), there are limited studies characterizing differences by LTBI status in circulating inflammatory markers, in the absence of antigen stimulation (6–8). This information could potentially explain why an increased risk of certain adverse outcomes (*e.g*. acute myocardial infarction) has been observed among LTBI+ individuals, or help identify immune profiles associated with TB progression (9, 10).

One hypothesis on levels of inflammation by LTBI status is that there is no difference in circulating inflammatory markers between LTBI+ and LTBI− individuals. *Mtb* infection is mainly quiescent during LTBI and can remain in this form for a long time without harm to most individuals (11, 12). However, recent data from studies in adults suggest that there might be differences in systemic inflammation by LTBI status (6–8, 13). For example, a study of Indian adults observed that after adjusting for potential confounders, LTBI+ individuals had significantly higher levels of circulating pro-inflammatory mediators IL-6 and MCP-1 but lower levels of C-reactive protein (CRP), another pro-inflammatory marker, compared to LTBI− individuals (6).

While studies have started to assess potential differences in systemic inflammation by LTBI status in non-pregnant adults (6–8, 13), there is no data on pregnant women. Pregnant women have a distinct immune profile compared to adults, and there are temporal changes in immunity during pregnancy (14). It is not currently known whether there is a difference in systemic inflammation between LTBI+ and LTBI− pregnant women, and how this might change by trimester of pregnancy. Furthermore, LTBI+ women have a higher risk of *Mtb* progression during pregnancy and *post-partum*, but the reasons are not clear (15–17). The immune profile during pregnancy, including the systemic inflammatory milieu, may inform on potential changes to immunity that increase susceptibility to TB disease during pregnancy. In order to address this research gap in our understanding of systemic immunity in LTBI+ pregnant women, we compared the levels of systemic inflammatory markers, at the second and third trimesters, by LTBI status in a cohort of pregnant women from Pune, India and explored the association of these immune markers with TB progression during pregnancy and post-partum.

## Methods

### Study Design and Population

A cohort study of pregnant women was conducted at Byramjee Jeejeebhoy Government Medical College (BJGMC) in Pune, India from 2016 to 2019. Adult pregnant women, aged 18–40 years and between 13 and 34 weeks of gestation (confirmed by early pregnancy ultrasound), receiving antenatal care at BJGMC were enrolled for this study. Pregnant women with active TB at entry were excluded. We enrolled four cohorts of pregnant women based on their latent tuberculosis infection (LTBI) and HIV status: 1) LTBI+HIV+ (N = 35), 2) LTBI+HIV− (N = 130), 3) LTBI−HIV+ (N = 44) and 4) LTBI−HIV− (N = 25). The sample size for this cohort was based on the primary objective of the cohort study which was to compare the concentrations of Th1 cytokines after MTB-specific antigen stimulation by stage of pregnancy. LTBI status was determined using Interferon Gamma Release Assay (IGRA Quantiferon TB-Gold) according to manufacturer’s instructions. Sampling within each cohort was based on convenience sampling of those that met eligibility criteria.

### Ethics Statement

All clinical investigations were conducted according to the principles expressed in the Declaration of Helsinki. Written informed consent was obtained from all participants. This study was approved by the institutional review boards and ethics committees at BJGMC, Johns Hopkins University, Weill Cornell and Columbia University. We followed guidelines for human experimentation from the US Department of Health and Human Services.

### Data Collection and Laboratory Procedures

Sociodemographic information and clinical data were collected from pregnant women at the enrollment visit (13–34 weeks of gestation), at the third trimester visit (for those enrolled in the second trimester), at delivery and approximately every 3 months *post-partum*. At each follow-up visit, women were administered a World Health Organization (WHO) TB symptom screening questionnaire. Women with a positive WHO symptom screen, unintentional weight loss since last visit or with clinical findings on examination were further investigated with sputum GeneXpert, acid-fast bacilli test, chest X-ray and abdominal ultrasound. Culture in Lowenstein Jensen (LJ) media and liquid Mycobacteria Growth Indicator Tube (MGIT) were performed for further confirmation in those with positive findings.

Relevant to this analysis, blood was also collected at each visit in heparin tubes and plasma samples were stored in −80°C until further use. We conducted single-plex immunoassays on second and third trimester plasma samples according to the manufacturer’s (R&D Systems, Minneapolis, MN) directions for soluble CD163 (sCD163), soluble CD14 (sCD14), intestinal fatty acid-binding protein (I-FABP), C-reactive protein (CRP), alpha 1-acid glycoprotein (AGP) and interferon-*β* (IFN*β*). The sensitivity of the assays were as follows: 0.613 ng/ml for sCD163, 125 pg/ml for sCD14, 6.21 pg/ml for I-FABP, 0.02 ng/ml for CRP, 0.54 ng/ml for AGP, and 50 pg/ml for IFN*β*. Multiplex immunoassays (Luminex assays from R&D systems) measuring IFN*γ*, Interleukin (IL)-1*β*, IL-6, IL-13, IL-17A and TNF were also performed on these samples. The sensitivity of the assays were as follows: 0.40 pg/ml for IFN*γ*, 0.80 pg/ml for IL-1*β*, 1.7 pg/ml for IL-6, 36.6 pg/ml for IL-13, 1.8 pg/ml for IL-17A, and 1.2 pg/ml for TNF. These markers were chosen based on their importance to TB, HIV and pregnancy outcomes. For Single-plex immunoassays, SpectraMax plate readers were used with SofMax Pro 6 software. Luminex xMAP technology MAGPIX platform was used for multiplex immunoassays with xPONENT software.

### Statistical Analysis

We combined the LTBI+ cohorts (HIV+ and HIV−) and LTBI− cohorts (HIV+ and HIV−) to study the relationship of LTBI status with second or third trimester inflammatory markers among 220 women with available inflammatory data. Differences in study population characteristics by LTBI status were determined using Fisher’s exact test for categorical variables and Wilcoxon rank-sum test for continuous variables. A p-value less than 0.05 was considered statistically significant and a p-value of less than 0.004 (0.05/12) was considered statistically significant after Bonferroni correction for multiple comparisons. We also compared median levels of each inflammatory marker, during the second and third trimester, between LTBI+ and LTBI− pregnant women using the Wilcoxon rank-sum test. Inflammatory markers were log_2_-transformed for the data to approximate normality.

We conducted univariable and multivariable linear regression to determine the change in log_2_concentrations of each inflammatory marker (outcome variable) by change in LTBI status (exposure variable), with separate cross-sectional analyses for markers measured in second trimester or third trimester. Multivariable models adjusted for age, mid-upper arm circumference (MUAC), HIV status, vegetarian diet and gestational diabetes status. We also tested models that further adjusted for smoking, education or preeclampsia. MUAC at the time of plasma sample collection (i.e. second or third trimester) was used in multivariable models as it is a more reliable indicator of nutritional status during pregnancy compared to body mass index. Sub-set analysis was performed using Wilcoxon rank-sum test to determine whether similar relationships between LTBI status and inflammatory markers were observed for only HIV-negative populations.

We also conducted an exploratory analysis, using univariable and multivariable logistic regression analyses, to determine whether third trimester inflammation levels (exposure variable) was associated with TB progression during pregnancy or post-partum (outcome variable). Progressors were defined as those who prospectively developed active TB after sample collection in third trimester and within study follow-up of one-year *post-partum*. We used STATA software version 15.0 for the data analysis.

## Results

### Study Population Characteristics

Our study population of pregnant Indian women (N = 220) had a median age of 23 years (interquartile range (IQR): 21–27) (**Table 1**). Only 25% had an education of less than secondary education, and 34% had an income below India’s poverty line (monthly income <10,255 Indian rupees). Around 28% of the women had a mid-upper arm circumference (MUAC) less than 23 cm [an indicator of undernutrition in pregnancy (18)] and 7% had an MUAC >30.5 cm, indicative of overweight (**Table 1**). Most of the women (88%) did not smoke, and 12% were vegetarians. Ten percent had gestational diabetes, and 11% had preeclampsia. As this cohort was stratified by HIV status, 32% of the pregnant women were HIV+ (all on antiretroviral therapy). Study population characteristics did not differ by LTBI status except for lower proportion of HIV (p-value <0.001) in LTBI+ women; as mentioned above, this was due to the stratified design of the study. LTBI+ women also had a lower proportion of gestational diabetes (p = 0.08) and less post-high school education (p = 0.09), but these differences were not statistically significant (**Table 1**).

**Table 1:** Characteristics of the study population (N = 220).

	Overall (N = 220)	LTBI+ (N = 155)	LTBI− (N = 65)	P-value
Age median (IQR)	23 (21–27)	23 (21–27)	24 (21–27)	0.51
Monthly Income Rs. 10,255 Rs. 10,255	75 (34) 143 (66)	51 (33) 103 (67)	24 (38) 40 (62)	0.54
Education None to primary Middle school to high school Post-high school	54 (25) 139 (63) 27 (12)	40 (26) 101 (65) 14 (9)	14 (22) 38 (58) 13 (20)	0.09
Mid-upper arm circumference <23 cm 23–30.5 cm >30.5 cm	62 (28) 143 (65) 15 (7)	48 (31) 97 (63) 10 (6)	14 (21) 46 (71) 5 (8)	0.37
Smoking status Yes No	26 (12) 194 (88)	20 (13) 135 (87)	6 (9) 59 (91)	0.50
Preeclampsia Yes No	25 (11) 195 (89)	18 (12) 137 (88)	7 (11) 58 (89)	0.99
Gestational Diabetes status Yes No	21 (10) 195 (90)	11 (7) 141 (93)	10 (16) 54 (84)	0.08
HIV Yes No	70 (32) 150 (68)	31 (20) 124 (80)	39 (60) 26 (40)	<0.001

Data are presented as number (%) of subjects unless otherwise stated. P-values were calculated using Fisher’s exact test for categorical variables and Wilcoxon rank-sum for continuous variables to determine the difference between LTBI+ and LTBI− pregnant women.

### Levels of Inflammatory Markers by LTBI Status

**Figure 1:**
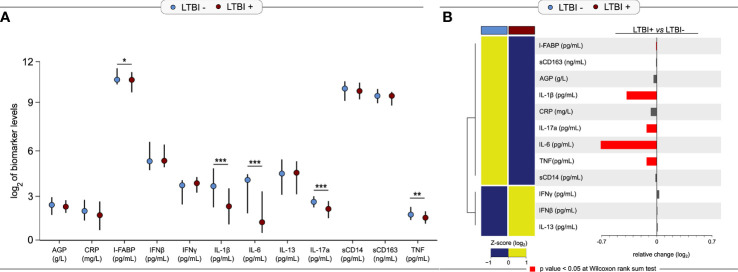
Levels of third trimester inflammation by LTBI status (N = 220). **(A)** Median and interquartile range (IQR) log_2_ levels of markers, measured in the 3^rd^ trimester is shown for LTBI+ (n = 155) and LTBI− (n = 65) pregnant women. Wilcoxon rank-sum test was used to calculate p-values. *p < 0.05, **p < 0.01 and ***p < 0.001. **(B)** Relative fold-change is shown for each marker by LTBI status. Red bars indicate p-value <0.05.

We compared the median log_2_-transformed levels of third trimester inflammatory markers by LTBI status using Wilcoxon-rank sum tests (**Figure 1**). IL-1*β* (3.64 *vs*. 2.25 pg/ml; p = 0.0002), TNF (1.76 *vs*. 1.54 pg/ml; p = 0.004), IL-6 (4.08 *vs*. 1.25 pg/ml; p< 0.0001) and IL-17a (2.48 *vs*. 2.16 pg/ml; p = 0.0001) were significantly higher in LTBI− women compared to LTBI+ women (**Figure 1**). IFN*γ* production upon *Mtb* antigen stimulation is used to define LTBI positivity; of note, IFN*γ* was lower (3.63 *vs*. 3.73 pg/ml; p = 0.15) in plasma (*i.e.* unstimulated samples) of LTBI− women compared to LTBI+ women, but this association was not statistically significant (**Figure 1**). Similar results were also observed when using log_2_ concentrations of markers measured in plasma samples from the second trimester (**Supplementary Figure 1**). LTBI− women had significantly higher levels of second trimester AGP, I-FABP, IL-1*β*, TNF, IL-6, and IL-17a compared to LTBI+ women (**Supplementary Figure 1**). LTBI− women also had lower levels of IFN*γ* compared to LTBI+ women, although this was not statistically significant (p = 0.08) (**Supplementary Figure 1**).

### Association of LTBI Status With Inflammation

**Figure 2:**
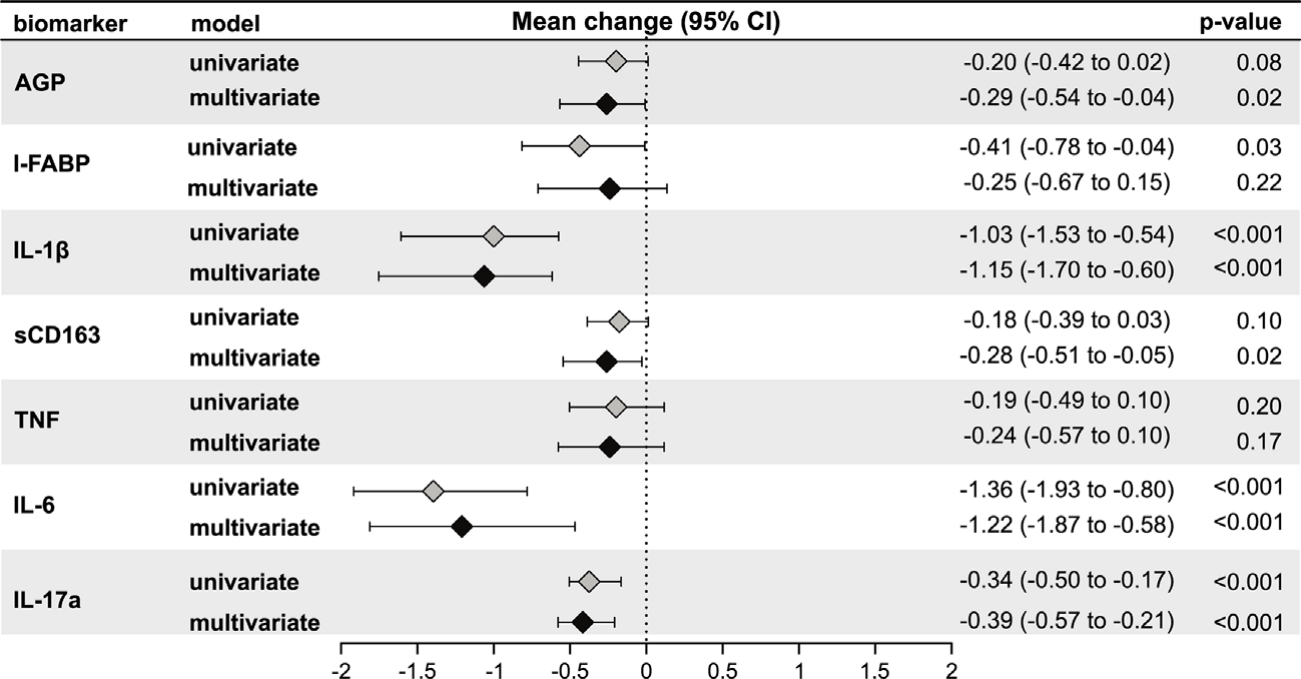
Association of LTBI status with third trimester inflammation (N = 220). Using linear regression, the mean change in log_2_ concentrations of each inflammation marker and 95% confidence intervals (95% CI) among LTBI+ individuals compared to LTBI−individuals are shown in the forest plot. Inflammation markers were measured in samples collected at the third trimester of pregnancy. Multivariate models adjusted for age, mid-upper arm circumference, HIV status, diet and gestational diabetes status. Only immune markers with a p-value <0.2 in the univariate model are shown.

Next, we assessed the relationship of third trimester inflammation with LTBI status using univariable and multivariable linear regression models. LTBI+ women had significantly lower levels of I-FABP (mean log_2_ change: −0.41, 95% confidence intervals (CI): −0.78 to −0.04; p = 0.03), IL1*β* (mean log_2_ change: −1.03, 95% CI: −1.53 to −0.54; p < 0.001), IL-6 (mean log_2_ change: −1.36, 95% CI: −1.93 to −0.80; p < 0.001), and IL-17a (mean log_2_ change: −0.34, 95% CI: −0.50 to −0.17; p <0.001) compared to LTBI− women in univariable models (**Figure 2**). AGP (mean log_2_ change: −0.20, 95% CI: −0.42 to 0.02; p < 0.08) and sCD163 (mean log_2_ change: −0.18, 95% CI: −0.39 to 0.03; p < 0.10) was also lower in LTBI+ women but this relationship was not statistically significant (**Figure 2** **)**.

After adjusting for age, third trimester MUAC, HIV status, vegetarian diet, and gestational diabetes in multivariable models, levels of IL-1*β* (mean log_2_ change: −1.15, 95% CI: −1.70 to −0.60; p < 0.001), IL-6 (mean log_2_ change: −1.22, 95% CI: −1.87 to −0.58; p < 0.001) and IL-17a (mean log_2_ change: −0.39, 95% CI: −0.57 to −0.21; p < 0.001), but not I-FABP (mean log_2_ change: −0.25, 95% CI: −0.67 to 0.15; p = 0.22), remained significantly lower in LTBI+ women compared to LTBI− women (**Figure 2**). In addition, AGP was also significantly lower in LTBI+ women (mean log_2_ change: −0.29, 95% CI: −0.54 to −0.04; p = 0.02) (**Figure 2**). After Bonferroni correction to adjust for multiple comparisons, third trimester IL1β, IL-6 and IL-17a were significantly lower in LTBI+ women in multivariable models.

Further adjusting for smoking, education or preeclampsia in multivariable models did not change the direction or significance of the results. Finally, we also conducted sensitivity analysis to show that when we limited the analysis only to HIV− subjects, the levels of these inflammatory markers were still lower in LTBI+ pregnant women compared to LTBI− women (**Supplementary Figure 2**), suggesting that HIV was not driving the observed relationships.

**Figure 3:**
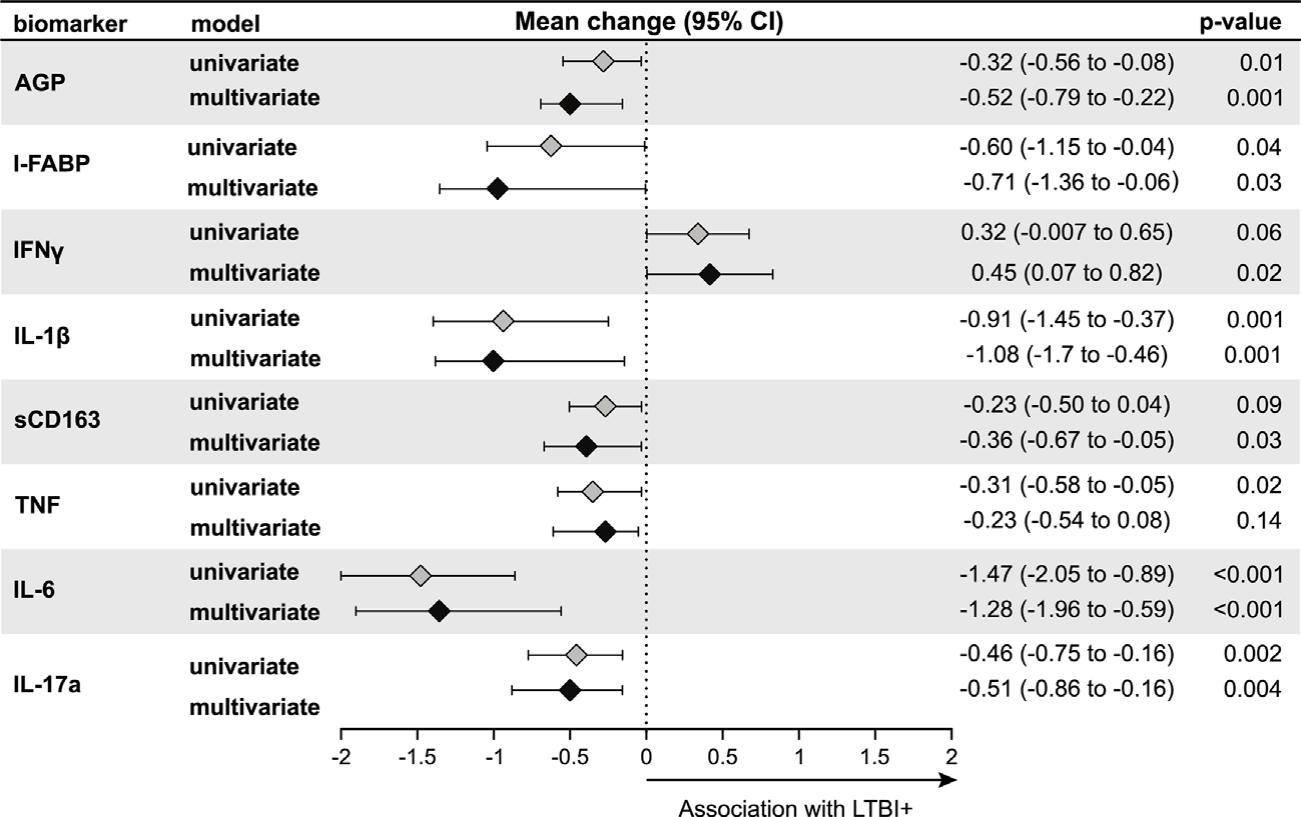
Association of LTBI status with second trimester inflammation (N = 187). Using linear regression, the mean change in log_2_ concentrations of each inflammation marker and 95% confidence intervals (95% CI) among LTBI+ individuals compared to LTBI− individuals are shown in the forest plot. Inflammation markers were measured in samples collected at the second trimester of pregnancy. Multivariate models adjusted for age, mid-upper arm circumference, HIV status, diet and gestational diabetes status. Only immune markers with a p-value <0.2 in the univariate model are shown.

Results using second trimester inflammatory markers instead of third trimester showed similar associations with LTBI status (**Figure 3**). In univariable models, LTBI+ pregnant women had significantly lower levels of AGP, I-FABP, IL1β, TNF, IL-6 and IL-17a compared to LTBI− pregnant women **(** **Figure 3**). In multivariable models, we observed similar results observed in univariable models with significantly lower levels of the AGP, I-FABP, IL-1*β*, IL-6, and IL-17a, but not TNF in LTBI+ compared to LTBI− women (**Figure 3**). In addition, sCD163 levels were significantly lower and IFN*γ* was significantly higher in LTBI+ women compared to LTBI− women (**Figure 3**). After Bonferroni correction to adjust for multiple comparisons, second trimester AGP, IL1*β*, IL-6 and IL-17a were significantly lower in LTBI+ women in multivariable models.

### Inflammatory Markers During Pregnancy and Progression of TB

**Figure 4:**
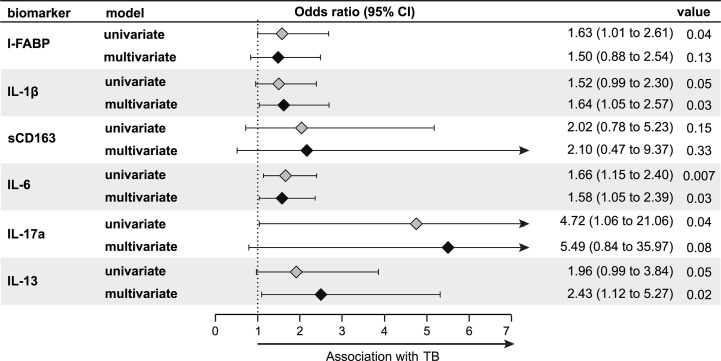
Association of third trimester inflammation markers with TB progression (N = 155; nine progressors). Using logistic regression, the odds ratio and 95% confidence intervals (95% CI) of TB progression per log_2_ increase in each inflammation marker among LTBI+ pregnant women are shown in the forest plot. Progressors were defined as those who developed TB either during the third trimester of pregnancy (n = 1) or up to one year *post-partum* (n = 8). Inflammation markers were measured in samples collected at the third trimester of pregnancy. Multivariable models adjusted for age, mid-upper arm circumference and HIV status. Only immune markers With a p-value <0.2 in the univariate model are shown.

We also conducted an exploratory analysis to test whether the systemic immune profile observed in LTBI+ pregnant women was associated with progression to active TB during pregnancy or post-partum. In our study, there were nine women, all LTBI+ at study baseline, who progressed to active TB either during the third trimester of pregnancy (n = 1) or *post-partum* (*i.e.* within one year of delivery) (n = 8). Given that all of the progressors were LTBI+ women, we present data comparing progressors and non-progressors only among LTBI+ women. Interestingly, levels of these markers in LTBI+ progressors, while higher than non-progressor LTBI+ pregnant women, were similar to LTBI− women (data not shown), suggesting that lower levels of these markers might be protective against TB progression in LTBI+ pregnant women. There was a significantly increased odds of progression per log_2_ increase in third trimester plasma levels of IL-1*β* (adjusted odds ratio (aOR): 1.64, 95% CI: 1.05–2.57), IL-6 (aOR: 1.58, 95% CI: 1.05–2.39), and IL-13 (aOR: 2.43, 95% CI: 1.12–5.27) after adjusting for age, MUAC and HIV status (**Figure 4**). There was also an increased odds for IL-17a (aOR: 5.49, 95% CI: 0.84–35.97), but this association was not statistically significant (**Figure 4**). Similar results were observed when we limited the analysis only to post-partum progressors (data not shown).

## Discussion

In our study of LTBI+ and LTBI− pregnant women from India, LTBI+ women had lower levels of various pro-inflammatory cytokines such as IL-1*β*, IL-6 and IL-17a compared to LTBI− women. In contract, the levels of IFN*γ* were higher (significant in second trimester) in LTBI+ women. While increased levels of IFN*γ* might be related to the use of this cytokine to define IGRA-based LTBI (19), the results with the other cytokines were surprising. These findings suggest that LTBI in pregnancy is characterized by a distinct immune profile with higher levels of IFN*γ* but lower levels of other immune markers with known roles in TB disease. Interestingly, LTBI+ women who progressed to active TB during pregnancy and *post-partum* did not have this profile in our exploratory analysis, suggesting the distinct immune profile in LTBI+ pregnant women might have a protective role against TB progression. Future larger studies will need to confirm these findings and determine whether these markers play a causal role and could be used to identify LTBI+ pregnant women at increased risk for TB progression and a target for preventative therapy.

LTBI+ pregnant women had significantly increased levels of IFN*γ* in the second trimester compared to LTBI− women. While the association was not statistically significant, the IFN*γ* levels were also higher for LTBI+ women in the third trimester. In our study, we used the IGRA test, which is dependent on IFN*γ* production (19), to define LTBI status; thus it might be expected IFN*γ* is higher in LTBI+ women. On the other hand, it should be noted that we measured IFN*γ* in plasma samples, and it is not obvious that IFN*γ* levels in circulation should also be higher for LTBI+ individuals. Our results here do indicate that higher levels of IFN*γ* are observed in circulation for LTBI+ pregnant women even without *Mtb* antigen stimulation. Similar results for IFN*γ* have also b-een observed from plasma samples of non-pregnant LTBI+ adults (13, 20). While the reasons are not clear, it is possible that despite being a latent infection, there could be periodic activity of some component (*e.g.* mRNA, protein) or low-level replication of *Mtb* that induces IFN*γ* production (13). Furthermore, LTBI is thought to be a spectrum of host–pathogen interactions, with ongoing replication and metabolic activity in certain subsets while quiescence in other *Mtb* subsets (9, 21).

Our data showed lower levels of immune markers, especially IL-1*β*, IL-6, IL-17a and AGP, in both trimesters, in LTBI+ women compared to LTBI− women. Higher levels of IFN*γ* can partly explain the lower levels of these other markers, as studies of *Mtb* have shown that IFN*γ* can have counteractive roles with IL-1*β*, IL-6 and IL-17a in certain instances (22–24). Pregnancy-specific changes in immune profile could also in part help explain these observations (14). For example, during pregnancy there is an increase in neutrophil levels (25, 26), which have been linked to lower levels of IL-6 and IL-17 in *Mtb* infection (1, 27).

Interestingly, in our exploratory analyses, LTBI+ TB progressors had a profile more similar to LTBI− women, with higher levels of IL-1*β*, IL-6, IL-13 and IL17a and generally lower levels of IFN*γ* compared to LTBI+ non-progressors. These inflammatory markers have been recognized for their complex role in TB disease where while a deficiency is linked to reduced control of *Mtb* infection, excessive levels can result in tissue damage and immunopathology (1, 28–33) as well as progression to active TB disease in non-pregnant adults (34). Given the small number of progressors in this study, these findings will need to be confirmed in other studies with a larger sample size. If these findings are confirmed, this profile could be used to identify subsets of LTBI+ pregnant women (*i.e.* those without this profile) at an increased risk of TB progression and would further support the idea of LTBI as a spectrum where subgroups of LTBI+ are protected from progression while others are not (9, 10). In addition, future studies would also need to determine whether this relationship of the systemic immune profile with TB progression is causal as it could partly explain the increased risk of *Mtb* progression during pregnancy and *post-partum* (15–17).

Our study has some limitations. We did not have data on inflammation markers from pregnant women during the first trimester or non-pregnant women. This data would be informative to understand whether the relationship of these markers with LTBI status was also similar in early pregnancy compared to later pregnancy, or in pregnant women compared to non-pregnant women. Regardless, our study did have longitudinal data on inflammatory markers in the second and third trimesters of pregnancy and showed consistent results with LTBI status in both trimesters that was robust to adjustments for multiple comparisons. Another limitation of this study is that we only assessed soluble markers of inflammation. The next steps for this study is to better understand the cellular sources of these differences by assessing potential differences in immune cell phenotype and function by LTBI status. The sample size for the analysis of TB progression was limited; while we were able to detect significant differences in multiple markers, this was an exploratory analysis that will need to be confirmed in larger studies. Future large studies should also address whether the changes in inflammatory markers due to LTBI status impacts the risk of birth and infant health outcomes.

In summary, we characterize the systemic immune profile in LTBI+ pregnant women showing higher levels of IFN*γ* but lower levels of other immune markers compared to LTBI− pregnant women. These findings describe a circulating cytokine and immune milieu indicating a distinct immune profile in LTBI+ women. Exploratory analysis suggests that this profile is negatively associated with TB progression. Future studies should confirm these findings in diverse settings in order to test the potential causal role along with the utility of this profile to identify women at high risk for TB progression and who may benefit from preventative therapy.

## Data Availability Statement

The raw data supporting the conclusions of this article will be made available by the authors, without undue reservation.

## Ethics Statement

The studies involving human participants were reviewed and approved by Johns Hopkins University; Columbia University; Weill Cornell Medicine; BJ Medical College. The patients/participants provided their written informed consent to participate in this study.

## Author Contributions

SN contributed to study design, implementation and interpretation. MA contributed to study design and interpretation and led the data collection. PK and SB conducted the laboratory assessments and contributed to interpretation of findings. VK and PD contributed to laboratory data collection and writing of this manuscript. SY and C-SL contributed to data analysis. MA-P and BA created the statistical scripts used to plot the analyses and graphs, and helped with the interpretation of findings. RB, AG, and JSM led the parent study and also contributed to the design, implementation and interpretation of this study. RS led the conceptual design, analysis and wrote the primary version of the manuscript. All authors contributed to the article and approved the submitted version.

## Funding

This work was supported primarily by the United States National Institutes of Health, NIH, Bethesda, MD, USA (R00HD089753 to RS and R01HD081929 to AG). JSM received support from NIAID (K23AI129854). Additional support for this work was the NIH-funded Johns Hopkins Baltimore-Washington-India Clinical Trials Unit for NIAID Networks (U01AI069465 to AG). BA is a senior investigator from the Conselho Nacional de Desenvolvimento Científico e Tecnológico (CNPq), Brazil. MA-P received a research fellowship from the Coordenação de Aperfeiçoamento de Pessoal de Nível Superior (CAPES; finance code 001). The content is solely the responsibility of the authors and does not necessarily represent the official views of the NIH.

## Conflict of Interest

The authors declare that the research was conducted in the absence of any commercial or financial relationships that could be construed as a potential conflict of interest.

The reviewer GW declared a past co-authorship with one of the authors BA to the handling editor.
