# Supplementary material for: Systemic Inflammation in Pregnant Women With Latent Tuberculosis Infection
Source: Front Immunol. 2021 Jan 27;11:587617. doi: 10.3389/fimmu.2020.587617 (PMC7873478; doi:10.3389/fimmu.2020.587617)
Supplement: Supplementary file 1 [file Table_1.docx]

Supplementary Material

# Supplementary Figure Title and Legends

**Supplementary Figure 1: Levels of second trimester inflammation by LTBI status (N=187)**

**Legend**: A) Median and interquartile range (IQR) Log_2_ levels of markers, measured in the 2^nd^ trimester is shown for LTBI+ (n=124) and LTBI- (n=58) pregnant women. Wilcoxon rank-sum test was used to calculate p-values. *p < 0.05, **p < 0.01 and ***p < 0.001. B) Relative fold-change is shown for each marker by LTBI status. Red bars indicate p-value < 0.05.

**Supplementary Figure 2: Levels of Inflammation by LTBI status in HIV- women in 3^rd^ trimester (N=139)**

**Legend**: A) Median and interquartile range (IQR) Log_2_ levels of markers, measured in the 3^rd^ trimester is shown for HIV- pregnant women with (n=124) and without (n=58) LTBI. Wilcoxon rank-sum test was used to calculate p-values. *p < 0.05, **p < 0.01 and ***p < 0.001. B) Relative fold-change is shown for each marker by LTBI status. Red bars indicate p-value < 0.05.
