# Supplementary figures and images for: Systemic Inflammation in Pregnant Women With Latent Tuberculosis Infection
Source: Front Immunol. 2021 Jan 27;11:587617. doi: 10.3389/fimmu.2020.587617 (PMC7873478; doi:10.3389/fimmu.2020.587617)

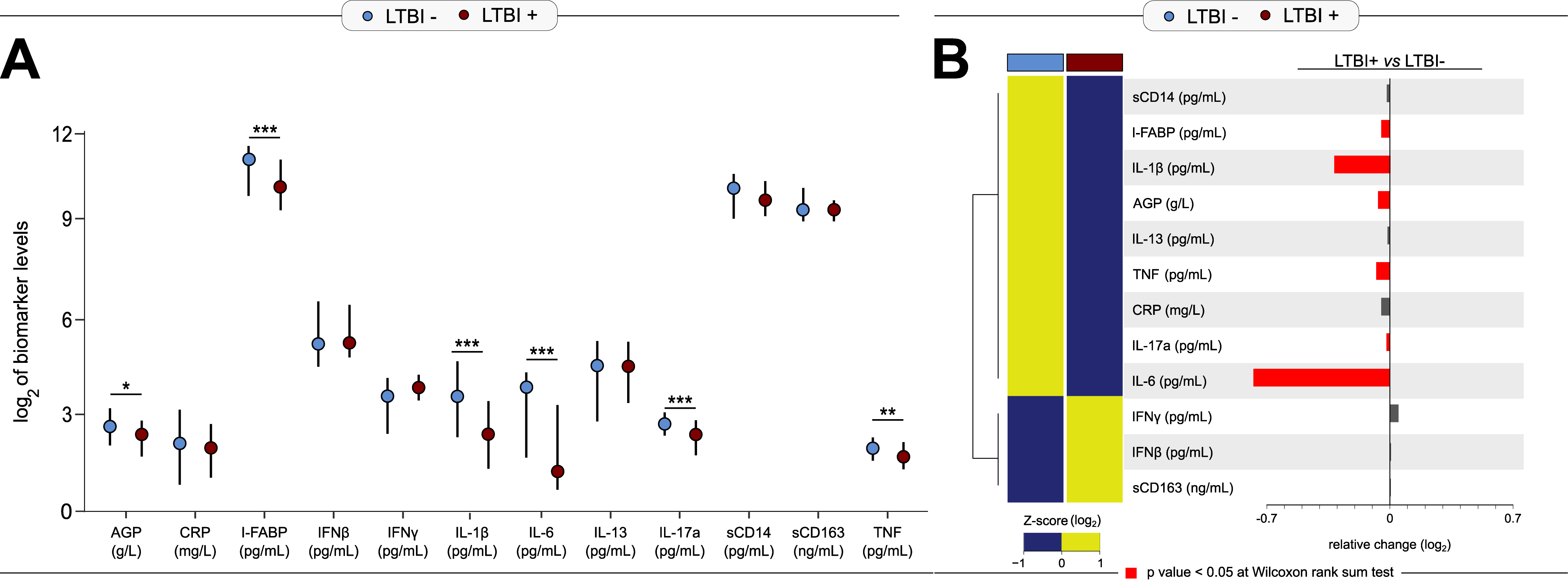

Supplement: Supplementary file 2 [file Image_1.tif]

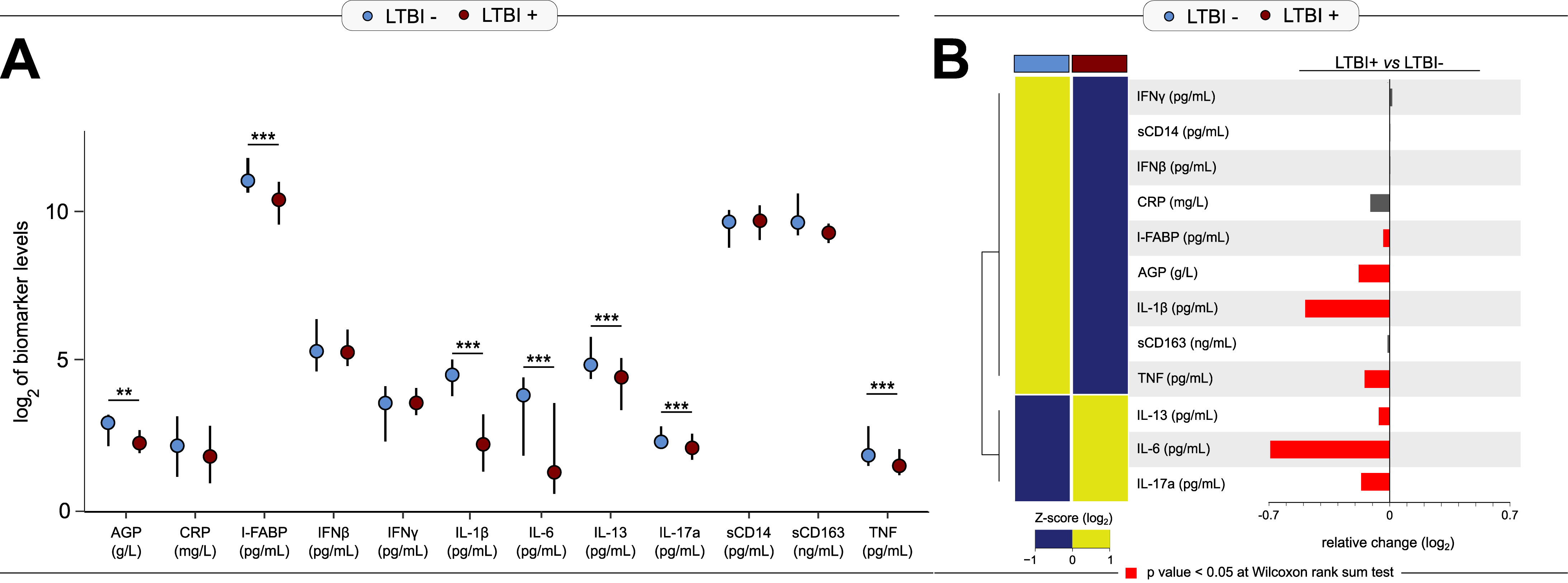

Supplement: Supplementary file 3 [file Image_2.tif]
